# Supplementary material for: Multivariate Gaussian Bayes classifier with limited data for segmentation of clean and contaminated regions in the small bowel capsule endoscopy images
Source: PLoS One. 2025 Mar 7;20(3):e0315638. doi: 10.1371/journal.pone.0315638 (PMC11888149; doi:10.1371/journal.pone.0315638)
Supplement: Attachment — Submitted filename: Response to Reviewers.docx [file pone.0315638.s002.docx]

**Manuscript Number: PONE-D-24-24094**

**Multivariate Gaussian Bayes classifier with limited data for segmentation of clean and contaminated regions in the small bowel capsule endoscopy images**

Dear academic editor, and reviewers,

We appreciate the time you have spent on our manuscript and thank for your constructive feedback and comments that helped us improve our study. We have answered all comments of the academic editor, and reviewers and corrected the mentioned issues. All answers to comments of academic editor, reviewer 1, and reviewer 2 have been highlighted in the text in green, red, and blue colors, respectively.

**Reply to academic editor’s comments**

We thank you for your insightful suggestions on enhancing our article. In response to the academic editor's comments, all revisions have been highlighted in green color within the manuscript.

| Comment 1 | Please ensure that your manuscript meets PLOS ONE's style requirements, including those for file naming. The PLOS ONE style templates can be found at  <https://journals.plos.org/plosone/s/file?id=wjVg/PLOSOne_formatting_sample_main_body.pdf> and  <https://journals.plos.org/plosone/s/file?id=ba62/PLOSOne_formatting_sample_title_authors_affiliations.pdf> |
| --- | --- |
| Reply | To address the academic editor’s comment regarding adherence to PLOS ONE's style requirements, we have tried the manuscript is formatted based on the guidelines specified in the provided links. This includes:   1. **File Naming**: We have checked all files to comply with PLOS ONE's naming conventions. 2. **Manuscript Formatting**: We have checked the manuscript layout, ensuring that it meets the requirements for font, margins, headings, and overall structure as outlined in the style templates. 3. **References and Citations**: We verify that all references are formatted correctly and consistently according to PLOS ONE standards. |
| Line number in the manuscript for correction | All lines |
| Comment 2 | Please note that PLOS ONE has specific guidelines on code sharing for submissions in which author-generated code underpins the findings in the manuscript. In these cases, all author-generated code must be made available without restrictions upon publication of the work. Please review our guidelines at <https://journals.plos.org/plosone/s/materials-and-software-sharing#loc-sharing-code> and ensure that your code is shared in a way that follows best practice and facilitates reproducibility and reuse. |
| Reply | Following the academic editor’s comment regarding code sharing in accordance with PLOS ONE guidelines, we carried out the following steps:   1. **Review Guidelines**: We carefully reviewed the PLOS ONE guidelines on code and data sharing to ensure compliance. 2. **Code Availability**: We prepared the author-generated code used in our study for public access, ensuring it is shared without restrictions in the GitHub repository. 3. **Repository Selection**: We selected GitHub and Figshare data repository to host the code and datasets, ensuring that they are easily accessible and well-documented. 4. **Documentation**: We provided clear documentation alongside the code, including installation instructions, usage examples, and any dependencies required for reproducibility. |
| Line number in the manuscript for correction | 599-604 |
| Comment 3 | In the online submission form, you indicated that [The three different datasets, and codes that support the findings of this manuscript are available from the first author (V.S.), upon reasonable request.].  All PLOS journals now require all data underlying the findings described in their manuscript to be freely available to other researchers, either 1. In a public repository, 2. Within the manuscript itself, or 3. Uploaded as supplementary information. This policy applies to all data except where public deposition would breach compliance with the protocol approved by your research ethics board. If your data cannot be made publicly available for ethical or legal reasons (e.g., public availability would compromise patient privacy), please explain your reasons on resubmission and your exemption request will be escalated for approval. |
| Reply | To address the editor’s comment regarding data availability, we have uploaded the **author-generated code** along with the **CECleanliness dataset** (containing images with their corresponding binary masks) to a publicly accessible GitHub repository, available at the following link: [<https://github.com/vahidsadeghi97/Clean-and-contaminatd-region-segmentation-in-the-WCE-images-using-GBC-model>]. Due to GitHub's storage limitations, we have hosted the remaining datasets — **Kvasir Capsule Endoscopy Dataset**, **SEE-AI Project Database**, and the **Degraded Kvasir Capsule Endoscopy Database** — on Figshare data repository. These datasets are publicly available and can be accessed with DOI: [<https://doi.org/10.6084/m9.figshare.27645021.v1>]  We hope this arrangement meets your expectation. |
| Line number in the manuscript for correction | 599-604 |
| Comment 4 | PLOS requires an ORCID iD for the corresponding author in Editorial Manager on papers submitted after December 6th, 2016. Please ensure that you have an ORCID iD and that it is validated in Editorial Manager. To do this, go to ‘Update my Information’ (in the upper left-hand corner of the main menu), and click on the Fetch/Validate link next to the ORCID field. This will take you to the ORCID site and allow you to create a new iD or authenticate a pre-existing iD in Editorial Manager. |
| Reply | Thanks for your comment. To address the academic editor’s comment regarding the ORCID iD for the corresponding author, we confirm that the ORCID iD is correctly displayed in the manuscript submission details. |
| Line number in the manuscript for correction | - |
| Comment 5 | Your ethics statement should only appear in the Methods section of your manuscript. If your ethics statement is written in any section besides the Methods, please delete it from any other section. |
| Reply | Following the academic editor’s comment concerning the placement of the ethics statement, we have reviewed the manuscript for any instances of the ethics statement outside the Methods section. We deleted any occurrences of the ethics statement that appear in sections other than Methods. Finally, we verify that the ethics statement in the Methods section is clear and appropriately detailed. |
| Line number in the manuscript for correction | 193-195 |

**Reply to reviewer 1’s comments**

We appreciate your valuable comments suggesting how to improve our article. Following the revision of the reviewer 1’s comments all answers have been marked in the manuscript in red color.

Reviewer 1's Responses to Questions

| Question 1 | Is the manuscript technically sound, and do the data support the conclusions?  The manuscript must describe a technically sound piece of scientific research with data that supports the conclusions. Experiments must have been conducted rigorously, with appropriate controls, replication, and sample sizes. The conclusions must be drawn appropriately based on the data presented. |
| --- | --- |
| Reviewer 1 response | Partly |
| Authors reply | We appreciate the reviewer’s comment. To ensure clarity and demonstrate the technical soundness of our study, we have elaborated on our experimental design, including details about controls, replication, and sample sizes. The following revisions have been made in the Results and Discussion sections:   1. **Experimental Design:** We have explicitly stated the image selection process for the datasets used (Kvasir, CECleanliness, and SEE-AI), detailing how images were annotated and how controls were established during the segmentation evaluations (line 27, 160, and 161 in the manuscript). 2. **Replication and Sample Size:** We clarified that our experiments were repeated five times for each dataset, with 20 annotated images selected as model construction subset for each trial (line 283-284, line 344-347, and line 413-415 in the manuscript). 3. **Data Support for Conclusions:** We emphasized the statistical significance of our results by considering metrics such as accuracy, Dice similarity coefficient (DSC), intersection over union (IOU), sensitivity, precision, specificity, and AUROC and ensured that conclusions are directly linked to the quantitative data. |
| Question 2 | Has the statistical analysis been performed appropriately and rigorously? |
| Reviewer 1 response | No |
| Authors reply | Thank you for your feedback regarding the statistical analysis in our manuscript. We recognize the incorporation of rigorous evaluation metrics, such as precision, recall, F1-score, and the area under the receiver operating characteristic curve (AUROC) can offer a more complete evaluation of the model's performance. These metrics are vital for understanding the trade-off between accurately detecting clean and contaminated regions, while also addressing challenges related to false positives and false negatives. We have calculated and added different performance metrics. |
| Question 3 | Have the authors made all data underlying the findings in their manuscript fully available?  The [PLOS Data policy](http://c05y1x9s.r.us-east-2.awstrack.me/L0/http:%2F%2Fwww.plosone.org%2Fstatic%2Fpolicies.action%23sharing/1/010f01920e62e163-e1cddc63-7df1-418d-9abd-79d4f86872fb-000000/t-qbGckJMRR9d5CVWcMGCsGTnXk=177) requires authors to make all data underlying the findings described in their manuscript fully available without restriction, with rare exception (please refer to the Data Availability Statement in the manuscript PDF file). The data should be provided as part of the manuscript or its supporting information, or deposited to a public repository. For example, in addition to summary statistics, the data points behind means, medians and variance measures should be available. If there are restrictions on publicly sharing data—e.g. participant privacy or use of data from a third party—those must be specified. |
| Reviewer 1 response | Yes |
| Authors reply | Thank you for your positive feedback on our data availability. We would like to inform you that, following the revision process, we have uploaded all relevant data and code to two public repositories on GitHub and Figshare data repository to ensure full compliance with the PLOS Data Policy.  Our GitHub repository now contains the CECleanliness dataset and the code used for the analysis and generation of results.  The repository is publicly accessible at the following link: [<https://github.com/vahidsadeghi97/Clean-and-contaminatd-region-segmentation-in-the-WCE-images-using-GBC-model>].  Given GitHub's storage limitations, the additional datasets — including the Kvasir Capsule Endoscopy Dataset, SEE-AI Project Database, and the Degraded Kvasir Capsule Endoscopy Database — have been hosted on Figshare data repository. These datasets are publicly accessible with DOI:   - Figshare data repository link: [<https://doi.org/10.6084/m9.figshare.27645021.v1>]   This ensures that all data and code are now fully available without restriction, in alignment with PLOS's open data requirements. |
| Question 4 | Is the manuscript presented in an intelligible fashion and written in standard English?  PLOS ONE does not copyedit accepted manuscripts, so the language in submitted articles must be clear, correct, and unambiguous. Any typographical or grammatical errors should be corrected at revision, so please note any specific errors here. |
| Reviewer 1 response | Yes |
| Authors reply | We sincerely thank the reviewer for his/her positive feedback regarding the clarity and language of our manuscript. |

Reviewer 1’s Comments to the Author:

| Comment 1 | The manuscript claims the model is novel, cost-effective, and suitable for resource-limited settings. This is highly relevant in medical applications where large annotated datasets are difficult to obtain. However, it would be helpful to elaborate on how this model differs from existing solutions. Can you provide a detailed comparison with other similar models to justify its applicability? |
| --- | --- |
| Reply | Thank you for your insightful feedback regarding the novelty and applicability of our model. We appreciate the opportunity to clarify how our model differs from existing solutions and to justify its relevance in resource-limited settings.   1. **Comparison with Existing Models:** We have expanded our discussion in the manuscript to include a detailed comparison of our model (GBC) with existing deep learning-based segmentation models, such as U-Net and Pix2Pix. This comparison highlights the following key differences:    - **Data Requirements:** Unlike deep learning models, which typically require large annotated datasets, our GBC model can achieve effective segmentation with significantly fewer annotated images due to its reliance on statistical modeling of color intensity values.    - **Computational Efficiency:** The GBC model is lightweight, containing only 26 parameters, making it suitable for deployment in low-resource environments without the need for high-performance GPUs.    - **Flexibility:** Our model's framework can be easily adapted for multi-class segmentation tasks, allowing for future expansion to include different types of lesions or other relevant features without a complete redesign of the model. 2. **Use Case Scenarios:** We have added specific scenarios in which our model can be particularly beneficial, such as:    - **Resource-Limited Settings:** Where access to extensive labeled datasets and high-performance computing resources is limited, our approach can be an optimal choice.    - **Rapid Implementation:** Since the GBC model in the model construction phase needs limited number of images, the GBC model can be implemented quickly, allowing for faster clinical integration and evaluation of bowel cleanliness in gastroenterology. 3. **Literature Support:** We have included references to studies and reviews that emphasize the challenges of obtaining large annotated datasets in medical imaging, supporting the need for our approach. We would like to highlight that the article entitled as "Semantic Segmentation Dataset for AI-Based Quantification of Clean Mucosa in Capsule Endoscopy," utilized 7,988 images from a total of 10,033 images to train a deep convolutional neural network model for the segmentation of clean and contaminated regions in the capsule endoscopy images. This demonstrates our model’s efficiency in achieving high performance with a relatively small dataset size, which is particularly important in resource-limited medical settings where large annotated datasets can be challenging to obtain. |
| Line number in the manuscript for correction | 500-504  509-513  516-517  526-533 |
| Comment 2 | Using various datasets to test the model's robustness and adaptability is commendable. The results show high accuracy and robustness across different datasets. However, a more detailed analysis of the characteristics of each dataset and how they influence the model's performance would be beneficial. What specific challenges did each dataset present, and how did the model overcome them? |
| Reply | Thank you for your valuable feedback on the robustness and adaptability of our model across various datasets. We agree that a more detailed analysis of each dataset's characteristics and their influence on model performance will enhance the manuscript. It is worth to mention that the SEE-AI project database, and CECleanliness dataset have been captured by the Pill Cam SB3 model, while the Kvasir capsule endoscopy database has been collected by using Olympus endocapsule. Since the captured images belong to two different brands, they differ from many aspects like image quality, resolution, and lighting conditions.   1. **Dataset Characteristics, challenges, and solutions:**    - **Kvasir Capsule Endoscopy Dataset:** This dataset contains images with varying degrees of contamination and diverse lighting conditions. The primary challenge here is the color variability due to differences in bowel contents, which can affect segmentation accuracy. The GBC model was able to adapt by utilizing prior knowledge of color intensity distributions to distinguish between clean and contaminated regions.    - **CECleanliness Dataset and SEE-AI Project Dataset:** The images in this dataset are characterized by high variability in illumination condition. The challenge lies in the different brightness capturing conditions. The GBC model effectively addressed this since both clean and contaminated regions can appear under different illumination conditions, constructing the Gaussian models on a variety of lighting conditions is helpful. The models can learn the variance in pixel values due to different lighting conditions, which improves generalization in the test phase. When the distributions account for different lighting conditions, the models can better distinguish color patterns based on intrinsic features of the clean or contamination regions, rather than just brightness or shadow effects. SEE-AI Project dataset and the CECleanliness database have different resolution compared to the Kvasir capsule endoscopy database. Since the GBC model consider the clean and contaminated regions segmentation as a pixel classification problem, it does not matter what is the resolution of the captured images, enabling the GBC model to generalize well across datasets with variable resolutions. |
| Line number in the manuscript for correction | 441-445  449-465 |
| Comment 3 | The manuscript highlights that while the GBC model may not achieve the highest accuracy compared to deep convolutional neural networks (DCNNs), it is significantly less resource-intensive. This point is critical, especially for clinical settings with limited computational resources. It would strengthen your argument to include detailed resource usage metrics (such as memory and computational time) for both the GBC and DCNN models. How do these differences impact practical applications in a clinical setting? |
| Reply | Thank you for this important observation. We appreciate the opportunity to clarify the resource usage metrics of our GBC model in comparison to deep convolutional neural networks (DCNNs).   1. **Resource Usage Metrics:**   **Memory Usage:** To calculate the storage space required to save a model, the following formula can be used:  Model Size (in bytes)=Number of Parameters×Size of Each Parameter  (in bytes)  Since we're using 32-bit floating point numbers (float32), each parameter will require **4 bytes** of storage. So,  Storage space (in bytes)=Number of parameters×4  Calculations for each model:   a) **GBC Model with 26 parameters**:   GBC storage space=26×4=104 bytes   b) **U-Net Model with 120,825 parameters**:   U-Net Storage space=120,825×4=483,300 bytes=483.3 KB   c) **Pix2Pix Model with 57,196,292 parameters**:   Pix2Pix Storage space=57,196,292×4=228,785,168 bytes=228.8 MB  The GBC model is designed to be lightweight, requiring approximately **104 Bytes** of memory, which is substantially lower than the typical **483 KB**, and **228.8 MB** required for the U-Net, and Pix2Pix architectures respectively. This significant difference makes the GBC model more feasible for deployment in settings with limited hardware capabilities.   2. **Impact on Practical Applications:**    a. **Suitability for Resource-Limited Settings:** The GBC model's low memory and computational requirements make it an ideal choice for clinical environments where access to high-end computational resources is limited. It can operate effectively on standard medical computers or even portable devices, ensuring broader accessibility. It is worth to mentioning, however, the training and evaluation of U-Net and Pix2Pix DCNNs have been carried out on the Google Colaboratory virtual machine with  an NVIDIA Tesla T4 GPU with 16 GB of VRAM (Video Random-Access Memory), the GBC model has been fitted and tested using an Intel Core i-5 CPU @ 2.50 GHz clock with 4.00 GB RAM.    b. **Efficiency in Clinical Workflow:** The reduced model construction time of the GBC allows for quicker decision-making in clinical settings.    c. **Focus on Cost-Effectiveness:** The affordability of deploying the GBC model aligns well with healthcare settings that often face budget constraints. By minimizing the need for expensive hardware, the GBC model enables healthcare facilities to implement advanced image analysis technologies without significant financial burden. |
| Line number in the manuscript for correction | Two last rows of Table 3  391-394  500-504  509-513  516-517 |
| Comment 4 | The process of selecting 20 images for training is not entirely clear. What criteria were used to ensure these images represented both clean and contaminated regions adequately? Providing a more detailed explanation of this selection process and ensuring the diversity of training images would address concerns about the model's generalizability. |
| Reply | Thank you for your insightful comment. We appreciate the opportunity to clarify the process of selecting limited number of images for training the model and ensuring that both clean and contaminated regions are adequately represented. Below is a more detailed explanation of the systematic selection process:  **Constructing the Training Dataset:**   1. **Initial Analysis of Color Distributions**: The article entitled as “Categorization and Segmentation of Intestinal Content Frames for Wireless Capsule Endoscopy” emphasizes the color patterns of the clean and contaminated regions in the capsule endoscopy images. The turbid fluids and bubbles are usually appeared in the predominant colors such as brown, yellow, green, and white. To ensure that both clean and contaminated regions are well represented in the training dataset, we first conducted a comprehensive analysis of color distributions across the entire dataset. This step helped us identify patterns in the images that are indicative of clean and contaminated regions. 2. **Feature Extraction and Clustering**: We converted the images into the LAB color space, which enhances the perceptual differences in color. From this, we calculated 3D color histograms, capturing the color distributions in the images. These histograms were flattened into feature vectors representing each image's color characteristics.   To group similar images, we applied the K-Means clustering algorithm to the resulting feature vectors. The number of clusters (K) was set to 20, ensuring the images were divided into 20 distinct categories based on their pixel intensity and color distributions. This step allowed us to categorize the images based on their visual color patterns, ensuring the inclusion of both clean and contaminated regions in each cluster.   3. **Image Selection**: From each of the 20 clusters, one image was randomly selected to serve as a representative of that cluster. This process ensured that the selected images adequately covered a diverse range of clean and contaminated patterns. 4. **Ensuring Representativeness**: By clustering the dataset and carefully selecting images from each cluster, we ensured that the training subset captured the pixel intensity distributions of both clean and contaminated regions. This diversity in the selected images is critical for the model's ability to generalize to unseen data and handle real-world variations in WCE images. 5. **Model Construction and Evaluation**: The 20 images selected from the clusters were used for model construction. The remaining 1980 images were set aside as the test subset, ensuring that the model was tested on a broad range of unseen images, further enhancing its robustness and generalizability across different clinical scenarios.   By following this structured approach, we ensured that the training dataset was not only diverse but also representative of the varying conditions found in clean and contaminated regions in WCE images. This process enhances the model's capability to generalize across different datasets and clinical situations, addressing concerns about its generalizability and robustness. |
| Line number in the manuscript for correction | 198-199  207-209  212-213  215-218 |
| Comment 5 | The manuscript should explain how the model deals with images that contain both clean and contaminated regions. Is there a specific threshold or rule applied in these cases? This is important because MDs do not drop contaminated images as they still may contain clinically relevant features. How does the model ensure that these features are not missed? |
| Reply | Thank you for your insightful comment regarding the handling of images that contain both clean and contaminated regions.  In our manuscript, we acknowledge the clinical relevance of images that may contain both types of regions. To address this, our model employs a probabilistic approach based on the Bayes rule. When processing images with mixed regions, the model classifies each pixel independently based on the learned pixel intensity distributions for clean and contaminated areas. Our rule-based technique is based on **Posterior probability method** explained in last paragraph of the “**Model construction and evaluation phases**” section. |
| Line number in the manuscript for correction | 278-284 |
| Comment 6 | The model's performance varies across different datasets. It would be insightful to discuss the potential reasons for these differences. Have you considered correlating the preparation scores of each image (like SBFVQ or BBPS) with the model's predictions? A subgroup analysis based on lesion type could also provide valuable insights into the model's performance in different clinical scenarios. |
| Reply | Thank you for your valuable comment regarding the variability of the model's performance across different datasets.  We acknowledge that the performance of the GBC model can vary depending on the characteristics of the dataset used. Several factors can contribute to these differences, including:   1. **Diversity of Image Quality**: Variations in the quality of the images across datasets impacts on model accuracy. For instance, datasets with a higher proportion of high-quality images may yield better segmentation results. 2. **Color Characteristics**: The inherent color distributions of clean and contaminated regions may differ between datasets. Our model performs better on datasets where the color spectrum of the clean and contaminated regions is more limited.   Correlation with Preparation Scores  We appreciate your suggestion to take into account the correlating preparation scores such as the Small Bowel Cleansing Quality (SBFVQ) or Boston Bowel Preparation Scale (BBPS) with the model's predictions. Although the BBPS has not been provided in the mentioned datasets, but we have calculated the SBVQ score between the ground truth labelled by our gastroenterologist and the GBC model. This correlation could provide deeper insights into how well the model performs in relation to established clinical evaluation metrics. This correlation has been discussed in the “**Comparison of SBVQ between the specialist and the GBC model**” section.  Subgroup Analysis  We sincerely appreciate your insightful suggestion to perform a subgroup analysis based on lesion type. We have indeed conducted such an analysis in our experiments, focusing on how the multivariate Gaussian Bayes Classifier (GBC) and deep convolutional neural networks (DCNNs) (U-Net and Pix2Pix) perform in segmenting clean and contaminated regions in capsule endoscopy images that include various lesion types.  Specifically, our subgroup analysis covered lesion types such as blood (both fresh and hematin), erosion, erythema, angioectasia, polyp, lymphangiectasia, and ulcer. The results demonstrated that the GBC and DCNN models performed well in classifying blood fresh, blood hematin, erosion, erythema, angioectasia, polyps, and most ulcers as part of the clean region. However, both GBC and DCNNs (U-Net and Pix2Pix) models encountered challenges in accurately segmenting lymphangiectasia and some samples of ulcers, primarily due to their color similarity to intestinal content, which led to their misclassification as part of the contaminated region.  We recognize that lymphangiectasia and some ulcers pose a more significant challenge because their color characteristics often resemble those of contaminating substances. In contrast, other lesion types exhibit more distinct visual color patterns, making it easier for the models to differentiate between clean and contaminated regions.  We agree that incorporating additional features, such as texture and shape analysis, may improve the model's segmentation performance, especially for lesions like lymphangiectasia and ulcer, where color similarity to contamination is problematic. Therefore, while the current color-based approach provides useful insights, we acknowledge that further work is required to address these challenging cases. In future studies, we plan to explore more advanced feature extraction methods to enhance the segmentation accuracy of clean and contaminated regions, particularly in the presence of visually similar lesions. |
| Line number in the manuscript for correction | 446-448  406-412  564-568 |
| Comment 7 | Including additional evaluation metrics such as precision, recall, and F1-score, AUROC, would provide a more comprehensive assessment of the model's performance. These metrics can help understand the balance between correctly identifying clean and contaminated regions, and addressing issues with false positives and false negatives. |
| Reply | Thank you for your insightful comment regarding the inclusion of additional evaluation metrics.  We agree that incorporating metrics such as precision, recall, F1-score, and area under the receiver operating characteristic curve (AUROC) would provide a more comprehensive assessment of the model’s performance. These metrics are crucial for understanding the balance between correctly identifying clean and contaminated regions while addressing potential issues with false positives and false negatives.  We have revised the manuscript to include a detailed analysis of these additional evaluation metrics. |
| Line number in the manuscript for correction | 28-31  Table 1, Table 2, and Table 3 |
| Comment 8 | Discussing the practical applications of this model in a clinical setting would be highly valuable. How do you envision this model being integrated into existing WCE review workflows? Current software already has functions for small bowel preparation scoring. How can this model enhance or complement these existing functions? Providing a roadmap for integration into clinical practice would demonstrate its potential real-world impact. |
| Reply | Thank you for highlighting the importance of discussing the practical applications of our model in a clinical setting.  We recognize that integrating our model into existing wireless capsule endoscopy (WCE) review workflows is crucial for demonstrating its real-world impact. However the capsule endoscopy reading software may be equipped with small bowel preparation scoring system, but these techniques work on the total image. In other words current capsule endoscopy reading software give a number to the whole image based on its visualization quality, but our GBC model works at the pixel level for segmentation purposes. The model could complement current software functions for small bowel preparation scoring in several ways:   1. **Enhancing Review Efficiency**: By automatically segmenting clean and contaminated regions, our model can reduce the time gastroenterologists spend reviewing video frames. This would allow them to focus on clinically relevant areas, improving overall efficiency. 2. **Complementing Existing Scoring Systems**: The model's statistical approach can provide an objective measure of bowel cleanliness alongside existing qualitative scoring systems. In contrast to the available capsule endoscopy reading software, the GBC model can be used for the segmentation task. 3. **Prioritizing Cases for Review**: The model could help prioritize which videos require more detailed examination based on the cleanliness scores generated, thus enabling gastroenterologists to allocate their time effectively. 4. **Training and Educational Use**: Our model could serve as a training tool for medical students and residents by providing feedback on the quality of bowel preparation, fostering better understanding and skills in evaluating WCE images.   We outline below the roadmap for integrating our clean and contaminated regions segmentation model into clinical practice. In clinical settings, the first step involves collecting capsule endoscopy images from patients undergoing routine procedures. These images are then automatically transformed into the LAB color space. The transformation enhances the separation between clean and contaminated regions by focusing on the luminance (L) and chromaticity (a and b) components.  The system computes 3D color histograms by dividing the L, a, and b components into 8 bins, forming a 512-dimensional feature vector for each image. By applying k-means clustering, representative images from each cluster are selected. The gastroenterologist must annotate the clean and contaminated regions in the selected images.  The system concatenates pixel intensity values of clean and contaminated regions separately, and computes the mean and covariance matrices for both regions. Two three-variate Gaussian Bayes classifiers are then fitted to distinguish clean from contaminated pixels. The simplicity of Gaussian models allows for efficient computation.  The trained model can be embedded into commercial capsule endoscopy reading software. Once deployed, the system processes each image, classifying pixels into clean or contaminated regions. This pixel classification enhances the quality of images analyzed by gastroenterologists by automatically filtering out obstructed regions (e.g., bubbles or turbid fluids).  The segmented images, with clear demarcations between clean and contaminated regions, can be displayed to gastroenterologists on dedicated software interfaces. The software can flag frames with significant contamination, allowing clinicians to focus on diagnostically relevant portions of the video stream, improving efficiency and reducing oversight in large image datasets.  After the procedure, the system can generate reports highlighting regions of interest while filtering out low-quality frames. This report could streamline the review process for clinicians, saving time and enhancing diagnostic accuracy.  By automating the identification of clean and contaminated regions, our technique has the potential to significantly reduce the time required by clinicians to review capsule endoscopy videos, improve diagnostic accuracy, and optimize workflow efficiency in clinical environments. |
| Line number in the manuscript for correction | 556-563 |

**Reply to reviewer 2’s comments**

We appreciate your valuable comments suggesting how to improve our article. All answers to reviewer 2’s comments (in the manuscript) have been marked in blue color.

Reviewer 2's Responses to Questions

| Question 1 | Is the manuscript technically sound, and do the data support the conclusions?  The manuscript must describe a technically sound piece of scientific research with data that supports the conclusions. Experiments must have been conducted rigorously, with appropriate controls, replication, and sample sizes. The conclusions must be drawn appropriately based on the data presented. |
| --- | --- |
| Reviewer 2 response | Yes |
| Authors reply | We sincerely thank the reviewer for acknowledging that the manuscript is technically sound and that the data support the conclusions. We have made every effort to ensure that the experiments were conducted rigorously, with appropriate controls, sufficient replication, and sample sizes. We carefully designed the experiments to assess the performance of the models, ensuring that the conclusions are well-supported by the data presented. |
| Question 2 | Has the statistical analysis been performed appropriately and rigorously? |
| Reviewer 2 response | Yes |
| Authors reply | We sincerely thank the reviewer for the positive feedback regarding the appropriateness and rigor of our statistical analysis. It is worth to mention that we have included additional evaluation metrics in the revised version of the article, such as specificity, sensitivity, precision, and the Area Under the Receiver Operating Characteristic Curve (AUROC). We believe that these enhancements will provide a more comprehensive evaluation of our models' performance. |
| Question 3 | Have the authors made all data underlying the findings in their manuscript fully available?  The [PLOS Data policy](http://c05y1x9s.r.us-east-2.awstrack.me/L0/http:%2F%2Fwww.plosone.org%2Fstatic%2Fpolicies.action%23sharing/1/010f01920e62e163-e1cddc63-7df1-418d-9abd-79d4f86872fb-000000/t-qbGckJMRR9d5CVWcMGCsGTnXk=177) requires authors to make all data underlying the findings described in their manuscript fully available without restriction, with rare exception (please refer to the Data Availability Statement in the manuscript PDF file). The data should be provided as part of the manuscript or its supporting information, or deposited to a public repository. For example, in addition to summary statistics, the data points behind means, medians and variance measures should be available. If there are restrictions on publicly sharing data—e.g. participant privacy or use of data from a third party—those must be specified. |
| Reviewer 2 response | No |
| Authors reply | We appreciate the reviewer’s comment regarding data availability. In compliance with the PLOS Data policy, we have made all three annotated datasets and the author-generated code publicly available in our GitHub and Figshare data repositories. This ensures that all data underlying the findings described in our manuscript can be accessed without restriction.  We have included a Data Availability Statement in the revised manuscript, specifying the location of the datasets and code. |
| Question 4 | Is the manuscript presented in an intelligible fashion and written in standard English?  PLOS ONE does not copyedit accepted manuscripts, so the language in submitted articles must be clear, correct, and unambiguous. Any typographical or grammatical errors should be corrected at revision, so please note any specific errors here. |
| Reviewer 2 response | Yes |
| Authors reply | We sincerely appreciate the reviewer’s positive feedback considering the clarity and intelligibility of our manuscript. We have thoroughly reviewed the manuscript for any typographical or grammatical errors and made necessary corrections to ensure that the language remains clear, correct, and unambiguous throughout. Thank you for your valuable insights, which have helped us improve the quality of our submission. |

Reviewer 2’s Comments to the Author:

Major concerns:

| Comment | Fig 7: The x and y axis's meaning is unclear. Please define the meaning of clean region. Does it refer to the ratio of clean area in the whole image? |
| --- | --- |
| Reply | Thanks for your preciseness. In Figure 7, the x-axis represents the clean region in the whole image as evaluated by the experienced gastroenterologist, while the y-axis indicates the predicted clean region as assessed by the Gaussian Bayesian Classifier (GBC) model. The term "clean region" refers to the ratio of clean pixels to the total number of pixels in each image. |
| Line number in the manuscript for correction | 412-417 |
| Comment | Line 339-404: how do the accuracy, DSC, and IOU look like if we apply U-net and Pix2pix algorithms that are trained on the Kvasir dataset to classify SEE-AI and CECleanliness data? If the authors see worse results, this will further help the author to reason that the GBC algorithm can be transferred to different datasets, especially for datasets with limited data where neural network-based algorithms won’t work. |
| Reply | Thank you for your valuable comment regarding the evaluation of U-Net and Pix2Pix models, which were trained on the Kvasir capsule endoscopy dataset, and their performance on the SEE-AI and CECleanliness datasets. In response, we conducted the experiments, and there was a decrease in accuracy, DSC, and IOU. This drop is likely because the DCNNs primarily learned the color and pattern characteristics specific to the clean and contaminated regions in the Kvasir dataset. The SEE-AI and CECleanliness datasets differ in imaging conditions and patient demographics, contributing to this performance decline. These findings further support the argument that the GBC algorithm is more adaptable across different datasets, especially in cases with limited data where neural network-based models may struggle. |
| Line number in the manuscript for correction | 518-519 |

Minor concerns:

| Comment | Line 215: ( = \|) and (\|) change to p( = \|) and (y=c\|) |
| --- | --- |
| Reply | Following your valuable comment, we have updated the notation for clarity. |
| Line number in the manuscript for correction | 233 |
| Comment | Line 342: Assessment ‘of ’ the performance …. |
| Reply | We appreciate the reviewer’s attention to detail. We have corrected the phrasing from "Assessment the performance…" to "Assessment the performance of… " as suggested. Thank you for your valuable feedback. |
| Line number in the manuscript for correction | 367 |
